# Widely targeted metabolomics analysis reveals differences in volatile metabolites among four *Angelica* species

**DOI:** 10.1007/s13659-024-00485-5

**Published:** 2025-01-02

**Authors:** Jiaojiao Ji, Lanlan Zang, Tingting Lu, Cheng Li, Xiaoxu Han, Soo-Rang Lee, Li Wang

**Affiliations:** 1 Present Address: Shenzhen Branch, Guangdong Laboratory for Lingnan Modern Agriculture, Genome Analysis Laboratory of the Ministry of Agriculture, Agricultural Genomics Institute at Shenzhen, Chinese Academy of Agricultural Sciences, No 7, Peangfei Road, Dapeng District, Shenzhen, 518120 China; 2 College of Life Science and Technology, Huazhong Agricultural University, Wuhan, 430070 China; 3 Zhengzhou Research Base, State Key Laboratory of Cotton Biology, School of Agricultural Sciences, Zhengzhou University, Zhengzhou, 450001 China; 4 State Key Laboratory of Crop Stress Adaptation and Improvement, School of Life Sciences, Henan University, Kaifeng, 475004 China; 5 Shenzhen Research Institute of Henan University, Shenzhen, 518000 China; 6 Department of Biology Education, College of Education, Chosun University, Gwangju, 61452 South Korea; 7 Kunpeng Institute of Modern Agriculture at Foshan, Foshan, 528200 China

**Keywords:** *Angelica*, Volatile metabolites, Chinese traditional medicine, Phylogeny

## Abstract

*Angelica* L. has attracted global interest for its traditional medicinal uses and commercial values. However, few studies have focused on the metabolomic differences among the *Angelica* species. In this study, widely targeted metabolomics based on gas chromatography-tandem mass spectrometry was employed to analyze the metabolomes of four *Angelica* species (*Angelica sinensis* (Oliv.) Diels (*A. sinensis*), *Angelica biserrata* (R.H.Shan & Yuan) C.Q.Yuan & R.H.Shan (*A. biserrata*), *Angelica dahurica* (Hoffm.) Benth. & Hook.f. ex Franch. & Sav. (*A. dahurica*) and *Angelica keiskei* Koidz. (*A. keiskei*)). A total of 698 volatile metabolites were identified and classified into fifteen different categories. The metabolomic analysis indicated that 7-hydroxycoumarin and Z-ligustilide accumulated at significantly higher levels in *A. sinensis*, whereas bornyl acetate showed the opposite pattern. Furthermore, a high correspondence between the dendrogram of metabolite contents and phylogenetic positions of the four species. This study provides a comprehensive biochemical map for the exploitation, application and development of the *Angelica* species as medicinal plants or health-related dietary supplements.

**Graphical Abstract:**

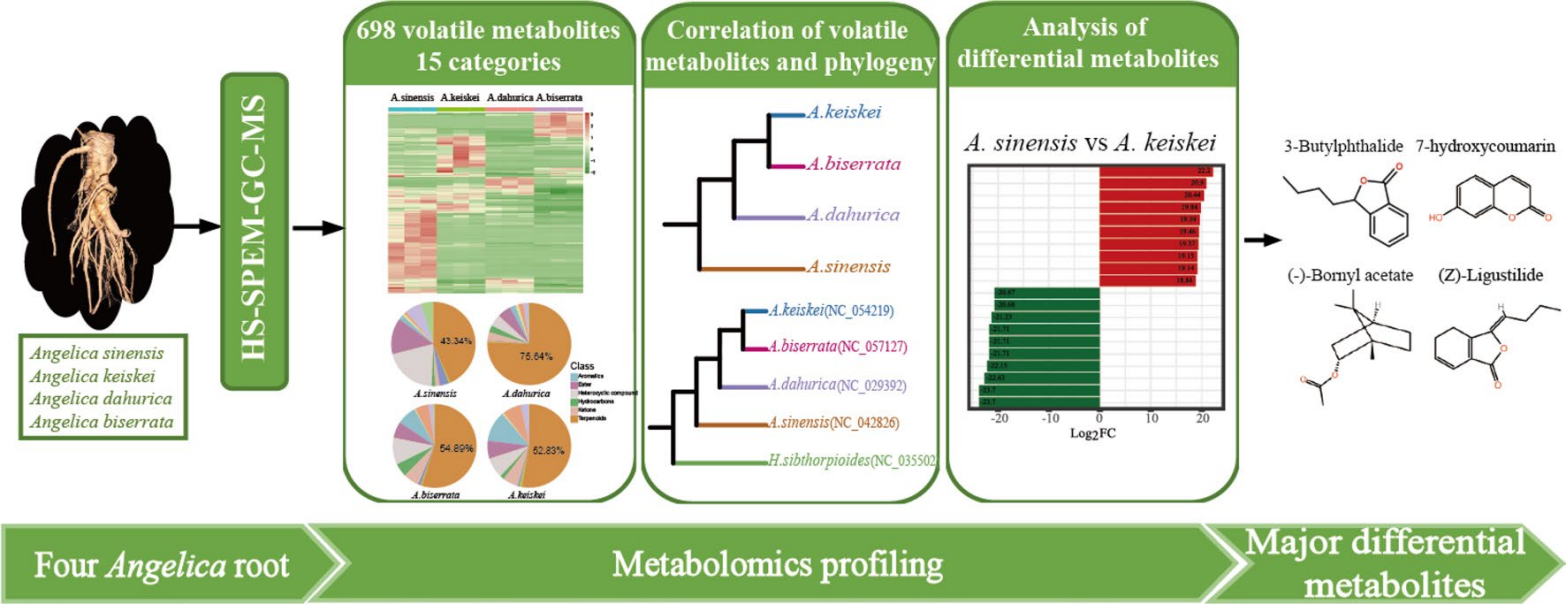

**Supplementary Information:**

The online version contains supplementary material available at 10.1007/s13659-024-00485-5.

## Introduction

*Angelica* L., a genus in the family Apiaceae, comprises 90 herbaceous species widespread in north-temperate regions, especially Eurasia [1, 2]. Many plants within the genus have long been used in traditional Chinese medicine (TCM) [3], especially the dried roots of *Angelica*, which are valued for their blood-nourishing, menstruation -regulating, and analgesic properties [1, 4]. Various herbal preparations containing *Angelica* species are available over the counter, not only in China, but also in Europe and American countries [5, 6]. Besides its medicinal value, *Angelica* species are also highly appreciated in various industrial applications such as the dietary supplements, perfumery, and cosmetics [1, 7, 8]. Previous studies have demonstrated that the pharmacological activity of aromatic and medicinal plants is attributed to its effective volatile components [9]. *Angelica* species are particularly rich in secondary metabolites, including coumarins, flavonoids, terpenoids, as well as volatiles oils (VOs) [1, 3]. VOs are complex mixture of low molecular weight volatile compounds isolated from the raw plant material through distillation [10], and they have been reported to treat serious health diseases, involving gynecological diseases, fever, and arthritis [1, 11]. Modern medical research has indicated that the VOs composition is mainly responsible for the medicinal properties of the *Angelica* genus [12]. For example, phthalides in *Angelica sinensis* (Oliv.) Diels (*A. sinensis*) are one of the highly effective VOs to analgesic and sedative activities [6, 13], while *Angelica biserrata* (R.H.Shan & Yuan) C.Q.Yuan & R.H.Shan (*A. biserrata*) contains active ingredients such as oxygenates, terpenoids, ketones and esters with analgesic and anti-inflammatory effects [14]. However, most current studies have focused on a limited number of targeted compounds within a single *Angelica* species. There is a lack of comprehensive comparative studies examining the volatile metabolites of multiple *Angelica* species, which poses a significant obstacle to the application and exploitation of these medicinal plants.

*A. sinensis*, known as female ginseng, is the most well-known medicinal plant within *Angelica* [15]. However, in terms of phylogenetic groups, *A. sinensis* belongs to the *Sinodielsia* clade, which is phylogenetically distant from core *Angelica* group [16]. In contrast, *Angelica dahurica* (Hoffm.) Benth. & Hook.f. ex Franch. & Sav. (*A. dahurica*) and *A. biserrata* are important TCMs located within core *Angelica* group, primarily used for expelling wind, removing dampness, relieving pain and treating inflammation [14, 17, 18]. A significant proportion of the compounds isolated from these two herbs are coumarins and volatile oils [18]. *Angelica keiskei* Koidz. has recently gained interest as a herbal medicine, dietary supplement, and health food in Asian countries [19]. It is known as “Myeong-Il Yeob” in Korea and “Ashitaba” in Japan, both of which mean “tomorrow’s leaf”. The leaves are used to make tea, which has been reported to lower blood pressure [20]. The medicinal parts of *A. sinensis*, *A. dahurica* and *A. biserrata* are roots, whereas that of *A. keiskei* are leaves. Additionally, these species have distinct geographical distributions. *A. biserrata*, *A. dahurica*, and *A. sinensis* are mainly found in China, while *A. keiskei* is predominantly distributed in Japan. Therefore, understanding the differences in the accumulation of medicinally important metabolites among these widely used species is crucial. This knowledge could provide a biochemical map for the development of unexploited wild species as medicinal plants or dietary supplements boosting health and may also facilitate the exploration of the evolutionary mechanisms underlying their biosynthetic pathways.

With the development of metabolomics, high-throughput and high-resolution methods such as headspace solid phase micro-extraction gas chromatography-mass spectrometry (HS–SPME–GC–MS) have been widely used to identify metabolite profiles and detect differences in the biochemical compositions of aromatic and medicinal plants [12, 21, 22]. In this study, volatile metabolites from four *Angelica* species *A. sinensis, A. dahurica*, *A. biserrata* and *A. keiskei* were identified and quantified using widely targeted metabolomics. The objective was to elucidate the differences in the accumulation of medicinally important metabolites among the four species. This study provides elucidate information on the chemical composition of *Angelica* plants and may facilitate the identification of the biologically active substances responsible for the pharmacological activity.

## Results

### Metabolomics profiling of four *Angelica* species

**Fig. 1:**
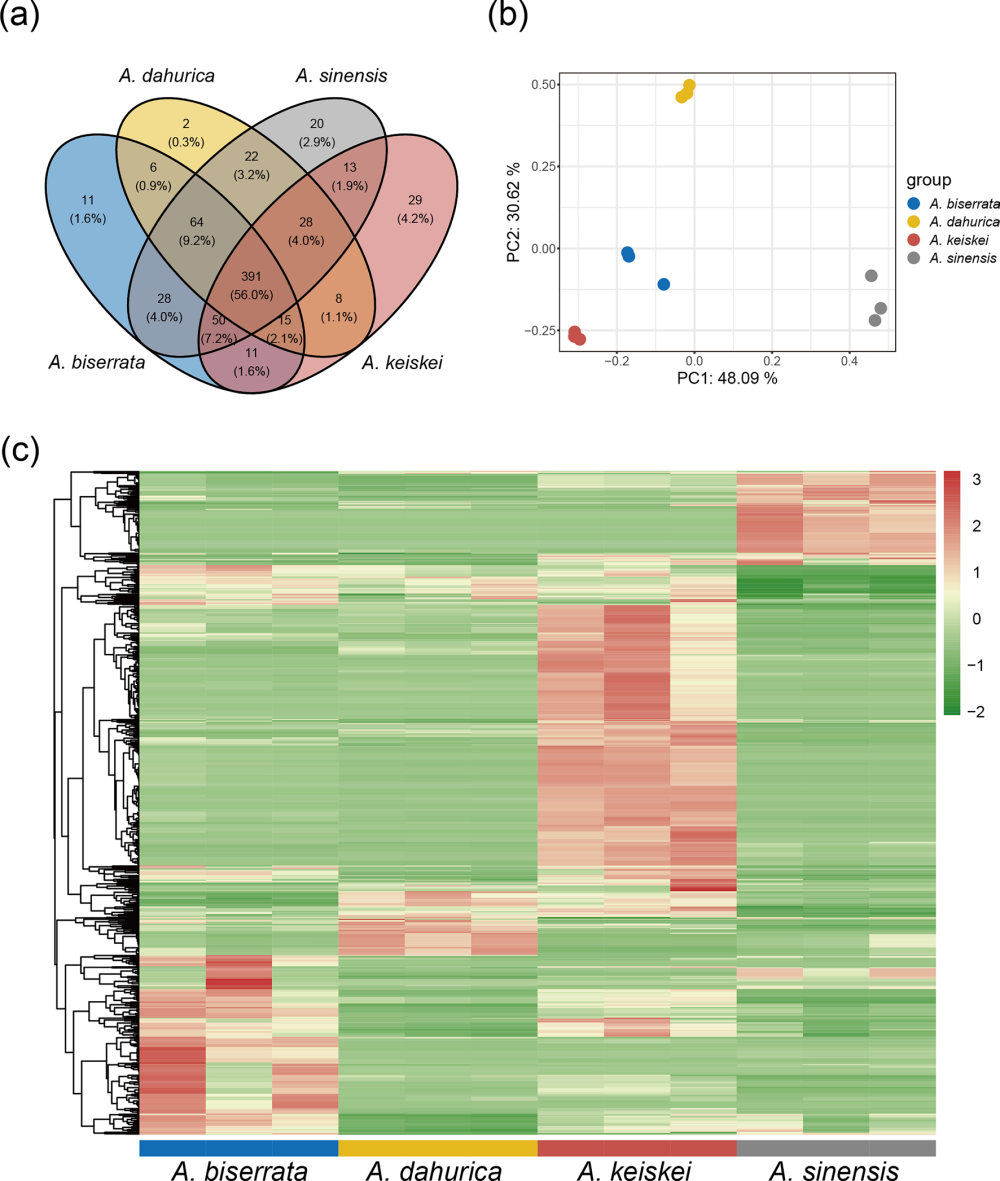
An overview of volatile metabolites among four *Angelica* species. **a** Venn diagram showing the number of common and specific metabolites in the four species. **b** PCA of volatile metabolites for the four species with three biological replicates. **c** Heatmap clustering of volatile metabolites identified from the four species. Volatile metabolite abundance was *Z-*score transformed. The color-coded scale grading from green to red corresponds to the content of volatile metabolites shifting from low to high

Widely targeted metabolomics provides a promising approach for the chemical screening of volatile metabolites, enabling the characterization of new metabolites in *Angelica* [12]. In this study, the root metabolomics data were generated to investigate the differences in volatile metabolites among four *Angelica* species. A total of 698 non-redundant volatile metabolites were qualified and quantified based on GC–MS (Table S1), with 616, 536, 576, and 545 metabolites detected in *A. sinensis*, *A. dahurica*, *A. biserrata*, *A. keiskei*, respectively. Of these, 391 metabolites were commonly detected in the roots of all four species (Fig. 1a).

Principal component analysis (PCA) of the metabolome data, transformed with Hellinger transformation method, divided the samples into four distinct groups corresponding to the four species. The three biological replicates were clustered closely, indicating high reproducibility and reliability. The PCA plot showed that PC1 and PC2 explained 48.09% and 30.62% of the total variance, respectively. Of the four clusters, PC1 mainly differentiated *A. sinensis* from the other *Angelica* species, while PC2 primarily segregated *A. dahurica* from the other *Angelica* species (Fig. 1b).

The abundances of volatile metabolites were normalized using *Z*-score and subjected to hierarchical clustering analysis (Fig. 1c). The results showed that significant differences among the four species. Of these, the abundance of volatile metabolites in *A. keiskei* was the highest.

### Identification of differential metabolites in four *Angelica* species

**Fig. 2:**
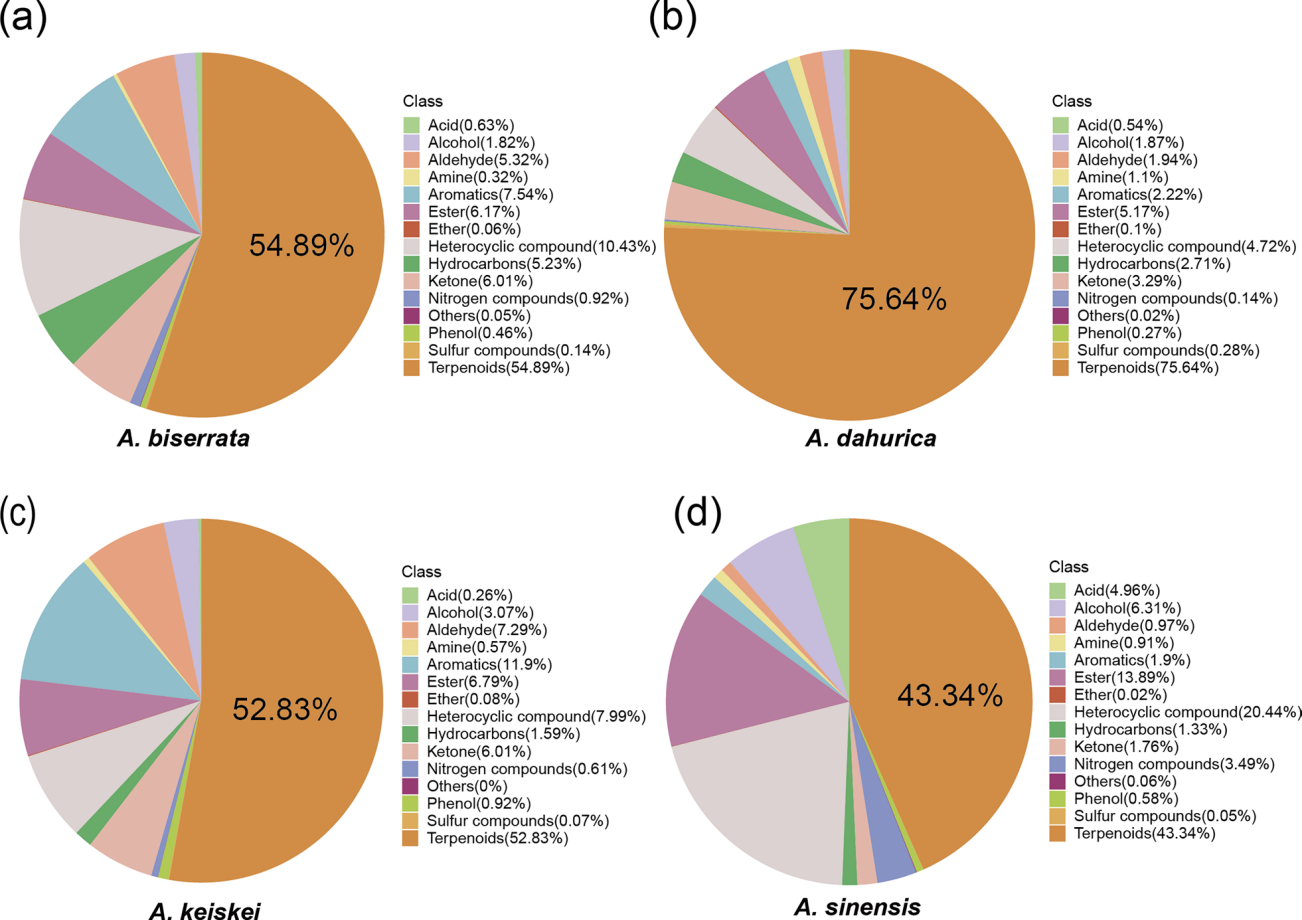
Classification and proportion of volatile metabolites detected in the four *Angelica* species. **a** *A. biserrata*, **b** *A. dahurica*, **c** *A. keiskei*, **d** *A. sinensis*

To explore the metabolite composition of the four species, 698 volatile metabolites were classified into 15 different categories, including terpenoids, ester, heterocyclic, aromatics and 11 others (Fig. 2). Terpenoids represented the largest proportion of all volatile metabolites across all four *Angelica* species, followed by heterocyclic compounds, eater, and aromatics. Notably, *A. sinensis* contained a relatively lower proportion of terpenoids (43.34%) compared to the other *Angelica* species, exhibiting a more balanced metabolite composition. In contrast, terpenoids amount accounted for over half of the total volatile metabolites in *A. dahurica*, *A. biserrata*, and *A. keiskei*, especially in *A. dahurica*, its proportion reached up to 75.64%.

**Fig. 3:**
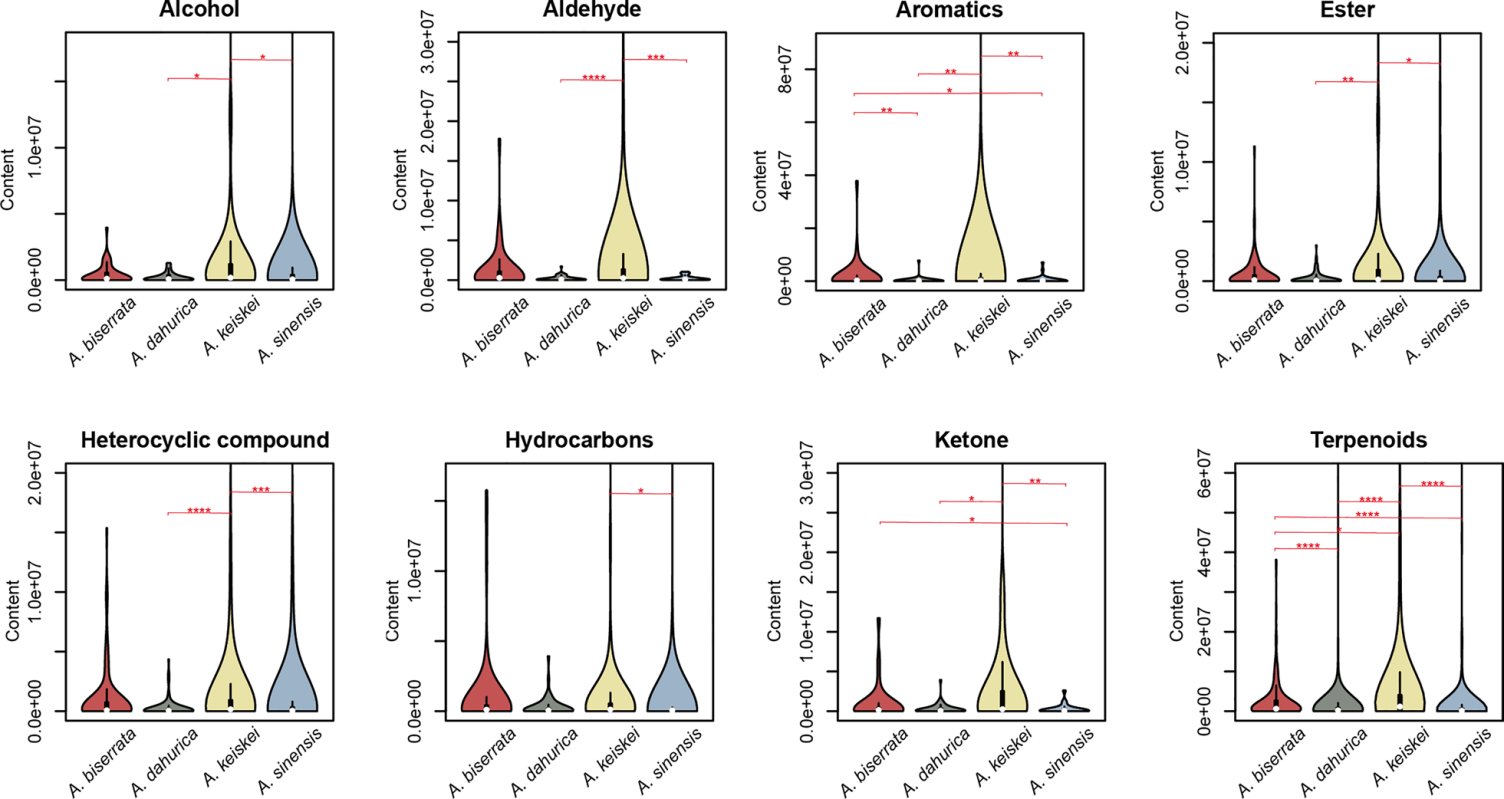
Comparison for the relative abundance of seven categories (alcohol, aromatics, aldehyde, ester, heterocyclic compounds, ketone and terpenoids) with significant differences in the four *Angelica* species

In addition, the relative abundance of each metabolite category in the four species were compared with Kruskal-Wallis test. Bonferroni-corrected *p*-values indicated significant difference in eight categories among the four species, including alcohol, aldehyde, aromatics, ester, heterocyclic compounds, hydrocarbons, ketone and terpenoids (Fig. 3, Figure S3; *p*-values were shown in the Table S2). Among these, terpenoids showed the most pronounced variation between species. In each of the eight categories, *A. sinensis* and *A. keiskei* differed significantly from at least one other species. These findings suggest that while the overall qualitative composition of volatile metabolites is similar, the individual components vary significantly between the *Angelica* species.

**Fig. 4:**
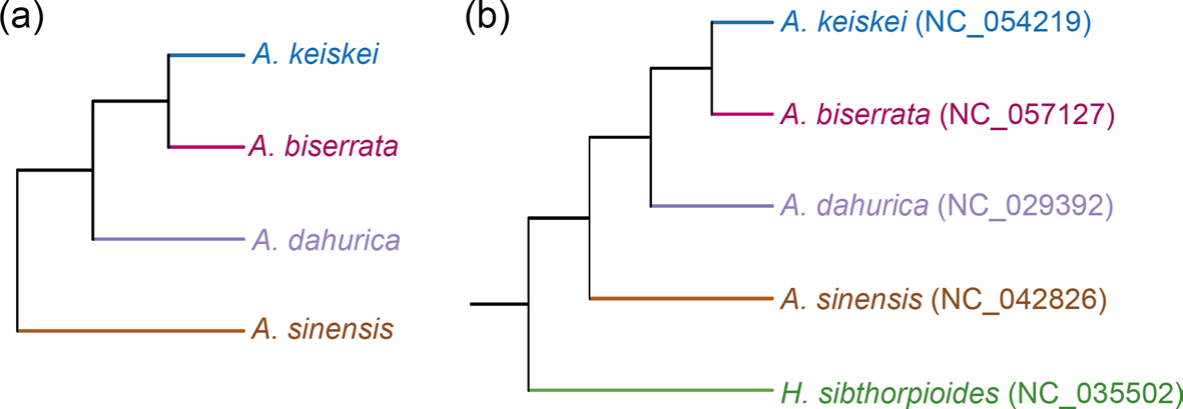
Hierarchical clustering based on the similarity of volatile metabolites (**a**) and phylogenetic tree of the four *Angelica* species and *H. sibthorpioides* (**b**). The chloroplast sequences above were available in GenBank of NCBI

In this study, hierarchical clustering analysis was conducted using Bray–Curtis’s dissimilarity distances to assess the composition and abundance of volatile metabolites across the four *Angelica* species. The resulting dendrogram (Fig. 4a) showed high correspondence with the phylogenetic tree (Fig. 4b) based on chloroplast sequences, indicating a correlation relationship between the volatile metabolites and the phylogenetic relationships.

### Differential metabolites between *A. sinensis* and the three other *Angelica* species

**Fig. 5:**
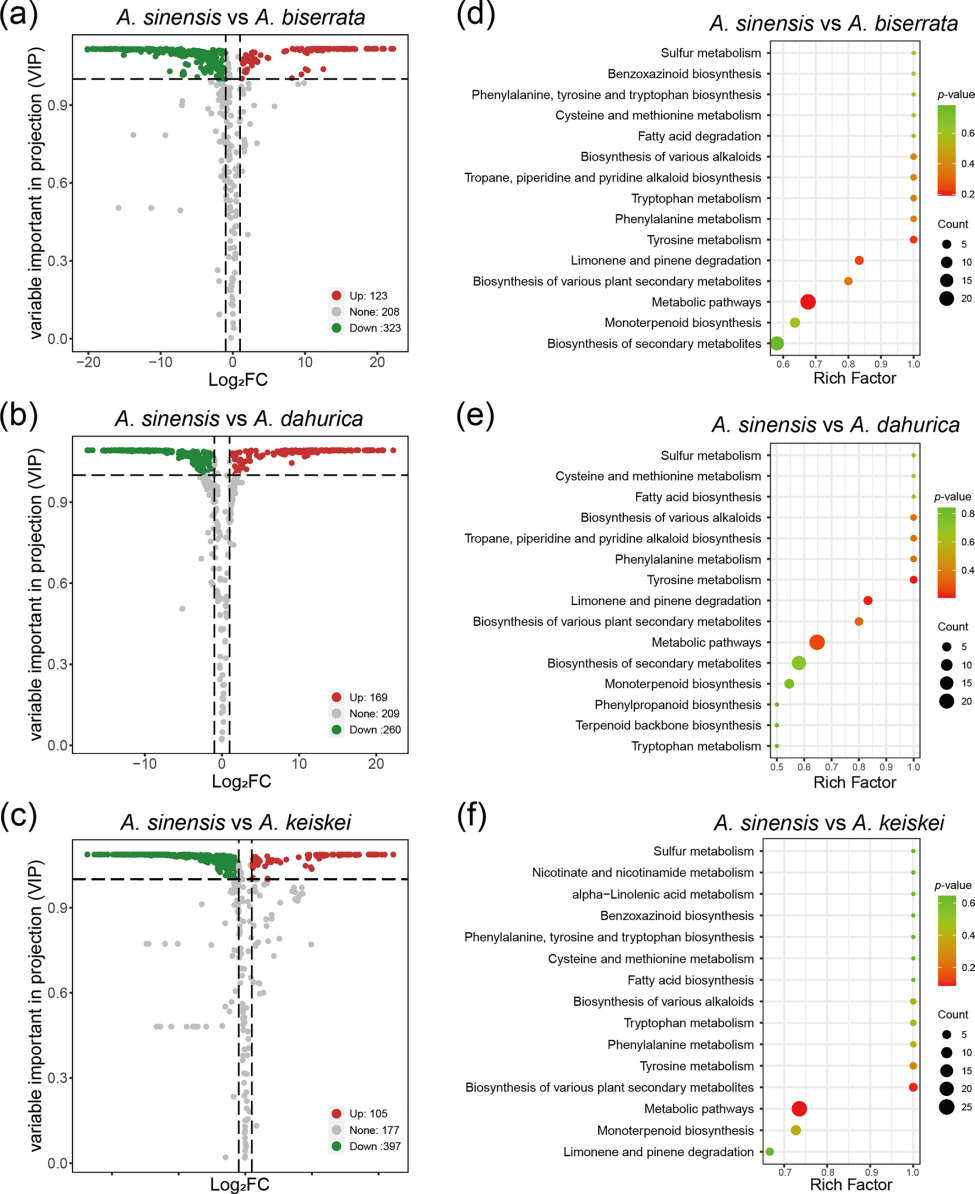
The overall distribution and KEGG enrichment analysis of differential metabolites between *A. sinensis* and the three other *Angelica* species. **a**–**c** Volcano plots for differential metabolites between *A. sinensis* and the three other *Angelica* species. **a** *A. sinensis* vs *A. biserrata*. **b** *A. sinensis* vs *A. dahurica*. **c** *A. sinensis* vs *A. keiskei*. Colors of metabolites indicated significant differences (red, upregulated; green, downregulated). **d**–**f** KEGG pathway enrichment analysis of differential metabolites for *A. sinensis* vs *A. biserrata* (**d**), *A. sinensis* vs *A. dahurica* (**e**) and *A. sinensis* vs *A. keiskei* (**f**). Color of the bubbles represented statistical significance of the enriched terms, and the size of the bubbles represented number of differentially enriched metabolites. The pathway of “Biosynthesis of various plant secondary metabolites” including: crocin biosynthesis, cannabidiol biosynthesis, mugineic acid biosynthesis, pentagalloylglucose biosynthesis, benzoxazinoid biosynthesis, gramine biosynthesis, coumarin biosynthesis, furanocoumarin biosynthesis, hordatine biosynthesis, podophyllotoxin biosynthesis

To further identify the metabolites responsible for differences among the four *Angelica* species, significantly different accumulated metabolites between groups were screened by |Log2FC|≥ 1 and VIP ≥ 1. *A. sinensis* belongs to *Sinodielsia* clade in *Angelica* genus, that was phylogenetically distant from core *Angelica* group, including *A. biserrata, A. dahurica*, *A. keiskei* [2, 23]. Moreover, PC1 mainly differentiated *A. sinensis* from the other three species (Fig. 1b), therefore, *A. sinensis* was used to comparison to other three species. Interestingly, *A. sinensis* exhibited fewer up-regulated metabolites compared to the other species. Additionally, no significantly enriched pathways were detected in the KEGG enrichment results of these differential metabolites, which could be a bias caused by the small dataset. In comparison to *A. biserrata*, 446 significantly differential metabolites were screened in *A. sinensis*, with 123 up-regulated and 323 down-regulated (Fig. 5a). The top 3 enrichment pathways were metabolic pathways (23 metabolites with *p* = 0.19), tyrosine metabolism (3 metabolites with *p* = 0.22) and limonene and pinene degradation (5 metabolites with *p* = 0.24) (Fig. 5d). When compared with *A. dahurica*, 429 significantly differential metabolites were detected in *A. sinensis*, including 169 up-regulated and 260 down-regulated (Fig. 5b). The top 3 enrichment pathways were tyrosine metabolism (3 metabolites with *p* = 0.21), limonene and pinene degradation (5 metabolites with *p* = 0.22) and metabolic pathways (22 metabolites with *p* = 0.26) (Fig. 5e). Finally, in comparison to *A. keiskei*, 502 significantly differential metabolites were identified in *A. sinensis*, with 105 up-regulated and 397 down-regulated (Fig. 5c), representing the largest number of differential metabolites among the groups. The top 3 enrichment pathways of these metabolites were metabolic pathways (25 metabolites with *p* = 0.09), biosynthesis of various plant secondary metabolites (5 metabolites with *p* = 0.10), and tyrosine metabolism (3 metabolites with *p* = 0.26) (Fig. 5f).

**Fig. 6:**
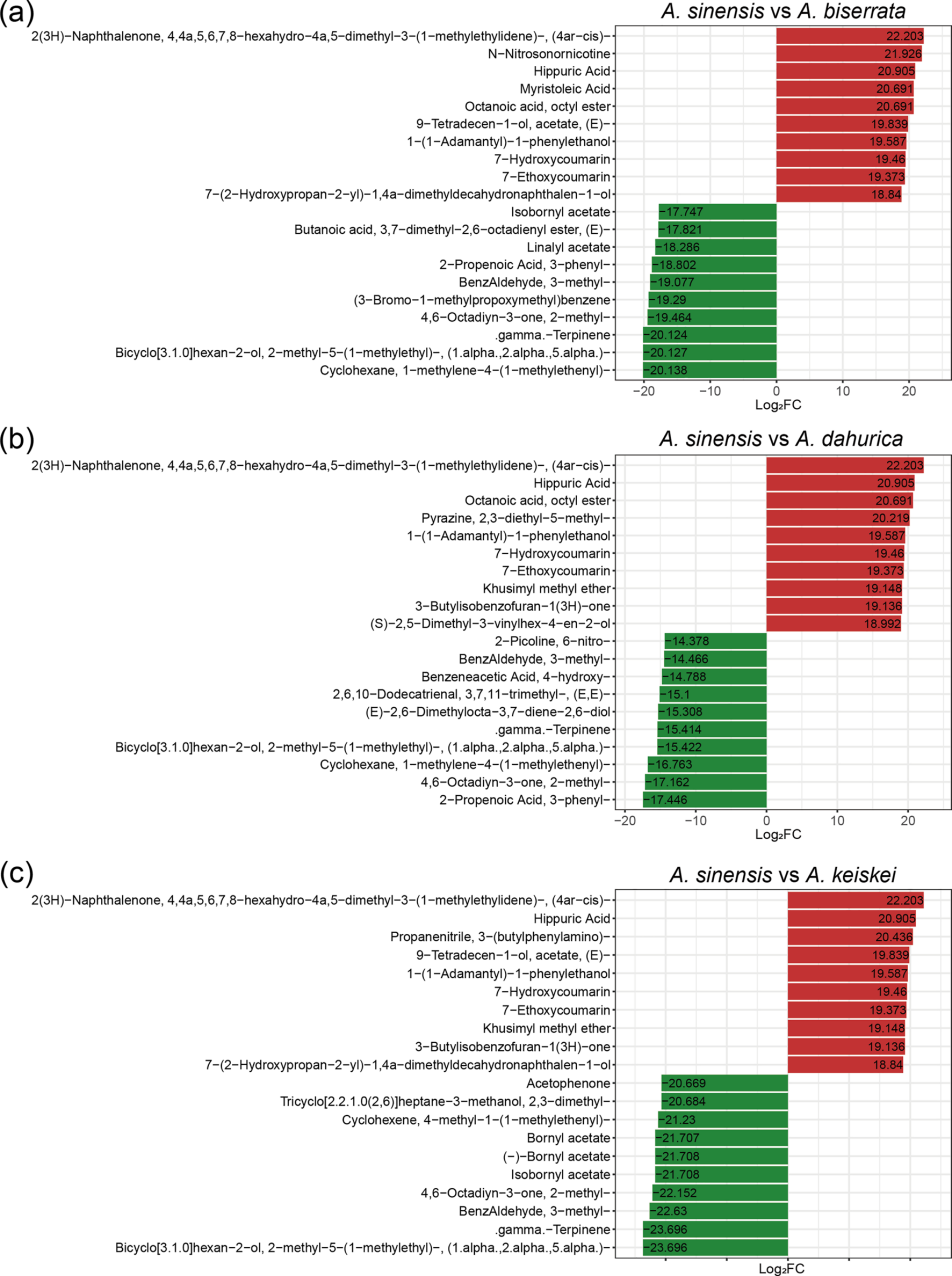
The top 20 metabolites of significantly differential volatiles between *A. sinensis* and three other *Angelica* species. Red indicates the more abundant metabolites in *A. sinensis* compared to *A. biserrata* (**a**), *A. dahurica* (**b**), *A. keiskei* (**c**). Green indicates the lower levels of metabolites in *A. sinensis* than that in other species

In order to delve into the details of the volatile metabolite difference between *A. sinensis* and the other three species, the most significantly twenty metabolites (the top 10 for up-regulation and down-regulation, respectively) were selected (Fig. 6). Hippuric acid, 7-hydroxycoumarin and 7-ethoxycoumarin were found to be more abundant in *A. sinensis* than the three other *Angelica* species*.* In addition, the abundance of 3-butylisobenzofuran-1(3H)-one was substantially higher *A. sinensis* than in *A. dahurica and A. keiskei* (log_2_FC > 19). In contrast, γ-terpinene and bornyl acetate were present in high abundance in *A. dahurica, A. keiskei* and *A. biserrata*, but were found in lower concentrations *A. sinensis*.

### Differential metabolites between *A. keiskei* and the three other *Angelica* species

**Fig. 7:**
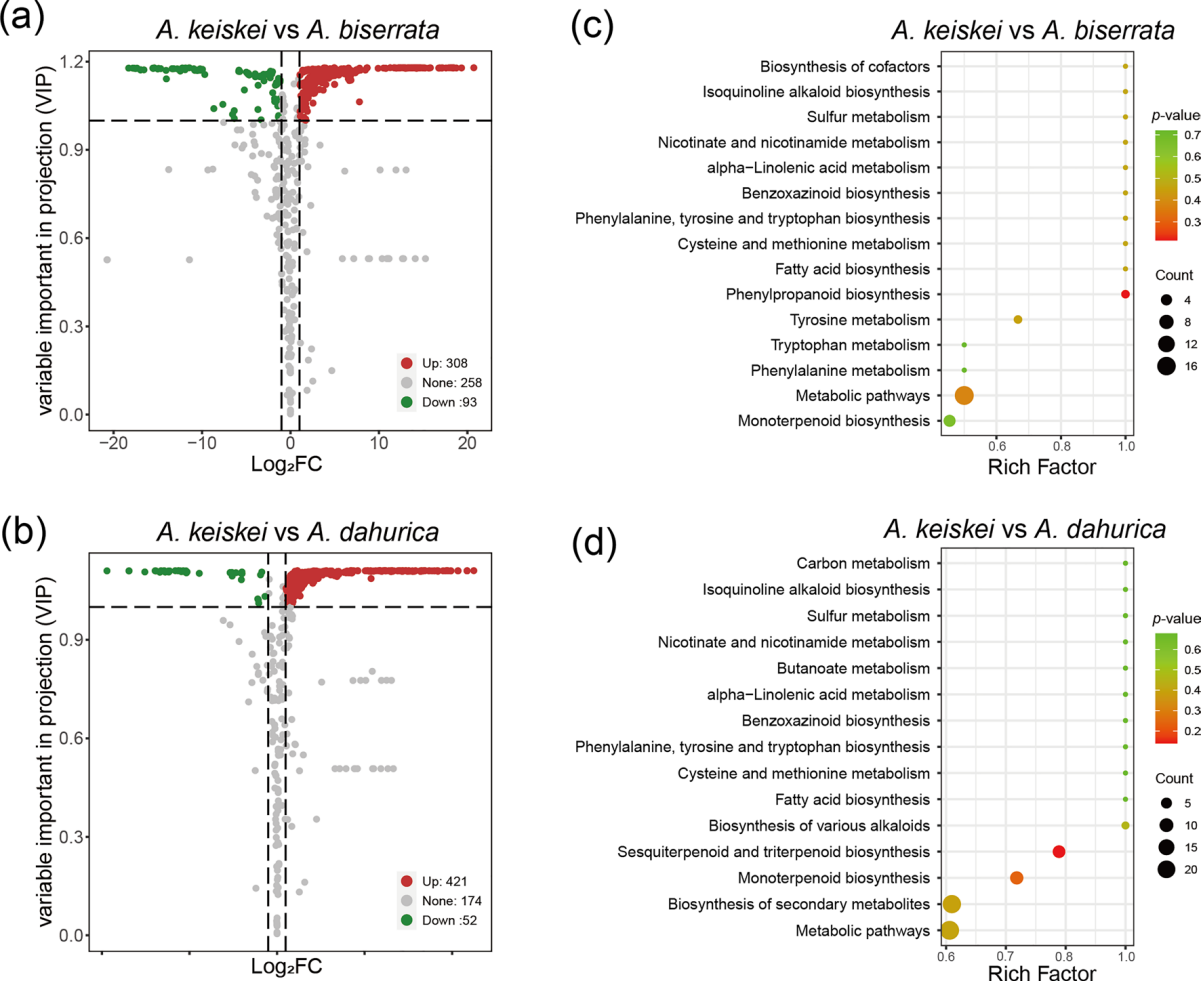
The overall distribution and KEGG enrichment analysis of differential metabolites between *A. keiskei* and *A. biserrata* (**a**, **c**), *A. keiskei* and *A. dahurica* (**b**, **d**). **a**, **b** Volcano plots for differential metabolites. The colors of metabolites indicated significant differences (red, upregulated; green, downregulated). **c**, **d** KEGG pathway enrichment analysis of differential metabolites. Color of the bubbles represented statistical significance of the enriched terms, and the size of the bubbles represented number of differential metabolites

The abundance of volatile metabolites in the roots of *A. keiskei* was the highest among the four species, suggesting that even the non-medicinal parts of *A. keiskei* have potential for practical applications. To further investigate, the differences of metabolites between *A. keiskei* and the other *Angelica* species were compared. The volcanic map visually showed the overall distribution of differential metabolites in each comparison. In the comparison between *A. keiskei* and *A. biserrata*, 401 significantly different metabolites were detected, with 308 up-regulated and 93 down-regulated (Fig. 7a). These metabolites were associated with phenylpropanoid biosynthesis (2 metabolites with *p* = 0.21), metabolic pathways (17 metabolites with *p* = 0.38) and tyrosine metabolism (3 metabolites with *p* = 0.45) (Fig. 7c). In the comparison between *A. keiskei* and *A. dahurica*, 473 significantly different metabolites were detected, with 421 up-regulated and 52 down-regulated (Fig. 7b), which were associated with sesquiterpenoid and triterpenoid biosynthesis (8 metabolites with *p* = 0.14), monoterpenoid biosynthesis (9 metabolites with *p* = 0.23) and biosynthesis of secondary metabolites (22 metabolites with *p* = 0.39) (Fig. 7d).

**Fig. 8:**
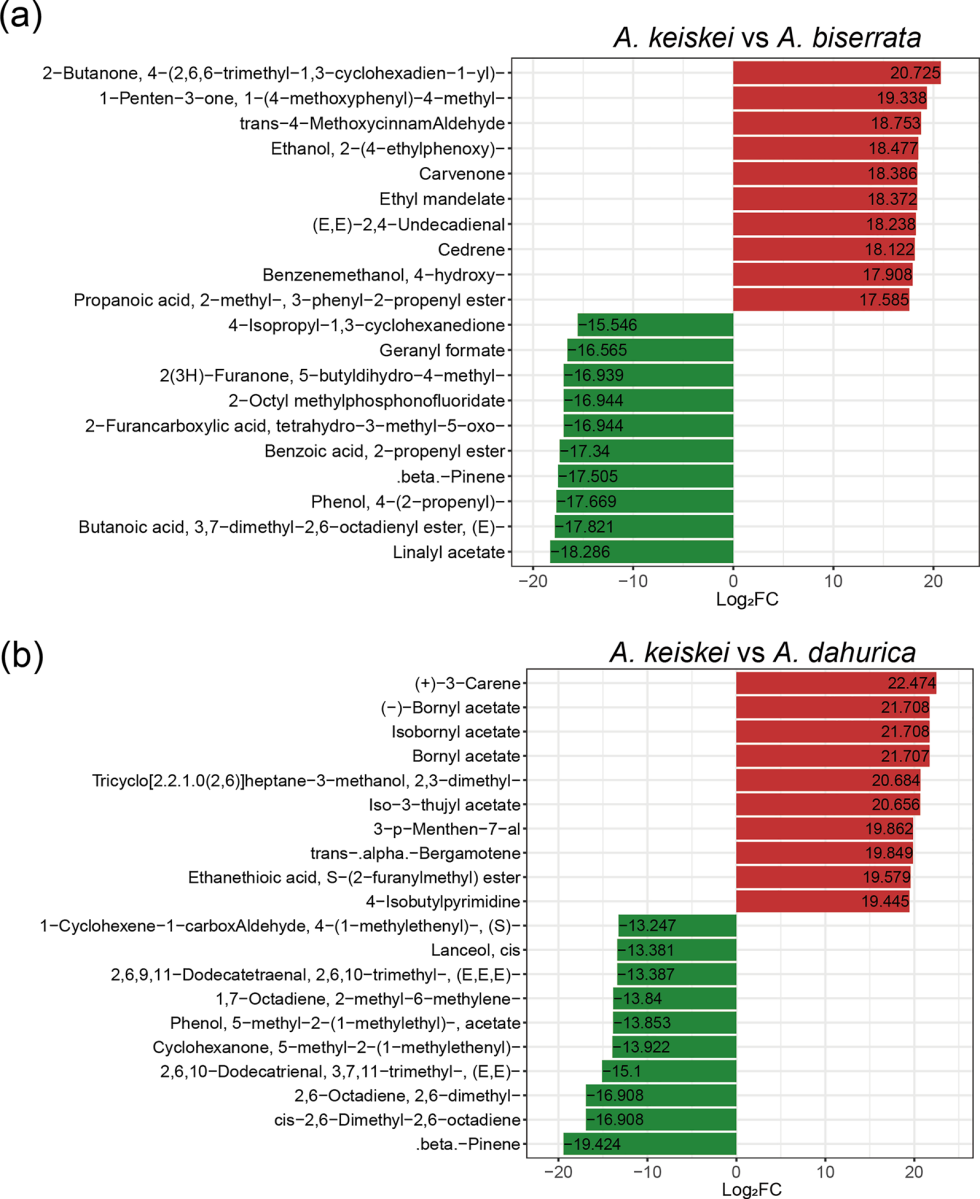
Top 20 metabolites with significant difference between *A. keiskei* and *A. biserrata* (**a**), *A. keiskei* and *A. dahurica* (**b**). Red and green represent up-regulated and down-regulated metabolites in *A. keiskei*, respectively

Moreover, to further investigate the differences of volatile metabolites between *A. keiskei* and other *Angelica* species, twenty of the most differentiated metabolites were selected for comparison (Fig. 8). The terpenoids metabolites, carvenone and cedrene were more abundant in *A. keiskei* than in *A. biserrate*, while carene, bornyl acetate and isobornyl acetate were the most enriched in *A. keiskei* compared to *A. dahurica*. Conversely, β-pinene was found in higher concentrations in *A. dahurica* and *A. biserrata* than in *A. keiskei.*

## Discussion

Widely targeted metabolomics offers a promising way for screening volatile metabolites and enables the identification of new volatile metabolites in *Angelica* [12]. Using the method, a total of 698 volatile metabolites were identified and classified into 15 categories, including terpenoids, ester, heterocyclic, aromatics and 11 others (Fig. 2). A clustering heat map of the metabolites revealed significant difference among the four species, with *A. keiskei* having the highest number and abundance of volatile metabolites. Consistent with the previous reports, terpenoids were the largest and most diverse class of volatile metabolites in the four *Angelica* species [1, 2]. Pairwise comparisons between species revealed fewer up-regulated metabolites in *A. sinensis* compared to the other three species (*A. dahurica, A. keiskei, A. biserrata*), while most differential metabolites were down-regulated in *A. dahurica and A. biserrata* relative to *A. keiskei.* These findings demonstrate that the analyzing differential metabolites is valuable for understanding the differences of chemical properties among the four species.

*A. sinensis* has long been used to treat various gynecological conditions [5, 6, 24], with phthalides believed to be responsible for its bioactive properties [6, 25]. This study found that 3-butylisobenzofuran-1(3H)-one and Z-ligustilide were present in all four species, with relatively higher concentrations in *A. sinensis*, consistent with previous studies [5]. In addition, coumarin and its derivatives, important heterocyclic metabolites [26], are known for their anti-HIV, anticancer activity agents, and anticoagulant activities [27, 28]. The results show that *A. sinensis* contained significantly higher levels of 7-hydroxycoumarin and 7-ethoxycoumarin than *A. dahurica*, *A. biserrata*, and *A. keiskei* (Fig. 2). Notably, 7-hydroxycoumarin, a precursor to furocoumarins and pyranocoumarins, plays a critical role in the biosynthesis of these compounds, which are key contributors to the herb's pharmaceutical activity [15, 29, 30]. Its higher abundance in *A. sinensis* was probably associated with this biosynthetic process. Nevertheless, in ancient Chinese medical systems, the pharmacological effect of medicinal plants depends not only on the high abundance of a single compound, but also on the synergy of multiple active ingredients [31, 32]. Furthermore, this study revealed that the proportion of various metabolites in *A. sinensis* was more balanced compared to the other species (Fig. 2), which may explain the widespread and versatile use in TCM.

Moreover, *A. keiskei*, known as ashitaba in Japanese, has traditionally been used for its leaves as herbs, food and spices [20, 33]. *A. keiskei* has been utilized both as a medicine and food due to its abundant pharmacological effects, including anti-cancer, lowering blood sugar and blood lipids, and improving human immunity [20, 34]. Currently, *A. keiskei* is primarily used in raw forms, such as tea and cosmetics, which limits its broader medicinal and clinical applications [33, 35]. Interestingly, bornyl acetate, a terpenoid previously unmentioned in *A. keiskei*, was detected with high expression levels in its root. Bornyl acetate is known for its antibacterial, insecticidal, and anesthetic effects, especially when combined with other aromatic metabolites in the VOs [36]. This finding supports the potential development of *A. keiskei* as a source of active ingredients for health-related dietary supplements. Overall, this study greatly enriches the chemical composition database of *A. keiskei* and suggests that its potential for medicinal and health-related applications.

Previous studies have verified that plants with closer phylogenetic relationship often exhibit similarities not only in morphology but also in chemical composition and curative effects [37–39]. This study indicated the high correspondence between the volatile metabolites and the phylogenetic relationships. Although more extensive sampling and deeper investigations would be necessary to establish more reliable correlations, the study implied that phylogenetic relationships could serve as a window to coarsely apprehend the unknown biochemical diversity of some plants based on the known biochemical map of phylogenetically related species. This finding may offer a great tool for searching replacements of medicinal plant resources that are endangered with closely related non-endangered species.

## Conclusions

This study investigated the metabolites of four *Angelica* species using widely targeted metabolomics and identified differences in the accumulation of medicinally important metabolites among species. For example, high levels of bornyl acetate metabolites were detected in *A. keiskei,* while coumarins and phthalides were significantly lower in *A. keiskei* compared to *A. sinensis.* Additionally, the high correspondence between the dendrogram of metabolite contents and the phylogenetic tree suggested a potential correlation between volatile metabolites and phylogenetic relationships. Taken all together, this study provides a comprehensive biochemical map for the exploitation, application, and development of the *Angelica* species as TCM or health-related dietary supplements.

## Experimental section

### Plant samples

Four species in genus *Angelica*, including *A. sinensis*, *A. dahurica*, *A. biserrata*, and *A. keiskei*, were analyzed in this study. The *A. sinensis* plants were collected in Minxian (104° 3′ 23″ E, 35° 2′ 16″ N), Gansu Province, China. The *A. dahurica*, *A. biserrata*, and *A. keiskei* plants were collected from the experimental field of the Agricultural Genomics Institute at Shenzhen (Chinese Academy of Agricultural Sciences, China). The specimens of the four species were deposited at Agricultural Genomics Institute at Shenzhen, Chinese Academy of Agricultural Sciences and identified by Prof. Li Wang (*A. sinensis* (202011002), *A. dahurica* (202008001), *A. biserrata* (202106003) and *A. keiskei* (202109004))**.**

Roots of each species were sampled with three biological replicates. The collected roots were washed, naturally dried, frozen in liquid nitrogen, and then stored at -80 ℃ for further analysis.

### Solid phase microextraction (SPEM) extraction

The samples were ground into powder in liquid nitrogen. Powdered samples (1 g) were weighed and transferred immediately to a 20 mL head-space vial (Agilent, Palo Alto, CA, USA), containing NaCl saturated solution to inhibit potential enzyme reactions. The headspace vials were sealed using crimp-top caps. As for SPME analysis, each vial was placed in 60 ℃ for 5 min, and then a 120 µm DVB/CWR/PDMS fiber (Agilent, Palo Alto, CA, USA) was exposed to the headspace of the sample for 15 min at 100 ℃. DVB/CWR/PDMS are three-phase fiber heads, which were confirmed to be able to extract more volatile metabolites than other fiber headers [40]. The quality control (mix) sample was prepared by mixing equal volumes of samples into a single tube, and during the instrumental analysis, a quality control sample was inserted into each of the 10 test samples to check the repeatability of the analysis process (Figure S1 and Table S1).

### GC–MS analysis

After the extraction procedure, the fiber was transferred to the injection port of the GC–MS system (Model 8890; Agilent, Palo Alto, CA, USA). The SPME fiber was desorbed and maintained in the injection port at 250 ℃ for 5 min in the split-less mode. The identification and quantification of volatile metabolites was carried out using an Agilent Model 8890 GC and a 7000 D mass spectrometer (Agilent, Palo Alto, CA, USA), equipped with a 30 m × 0.25 mm × 0.25 μm DB-5MS (5% phenyl-polymethylsiloxane) capillary column. Helium was used as the carrier gas at a linear velocity of 1.2 mL/min. The injector temperature was kept at 250 ℃ and the detector at 280 ℃. The oven temperature was programmed as followings: 40 ℃ (3.5 min), increasing at 10 ℃ min^−1^ to 100 ℃, 7 ℃ min^−1^ to 180 ℃, 25 ℃ min^−1^ to 280 ℃ and hold for 5 min. Mass spectra was recorded in electron impact ionization mode at 70 eV. The quadrupole mass detector, ion source and transfer line temperatures were set, respectively, at 150, 230 and 280 ℃. For the identification and quantification of analytes, the MS was selected ion monitoring mode.

### Qualitative and quantitative analysis

The qualitative analysis of primary and secondary mass spectrometry data, as well as the quantification of widely targeted metabolites, was performed by MetWare Biotechnology (Wuhan, China) with the self-built database MWDB and publicly available metabolite databases. Following the mass spectrometry analysis, all raw data were processed with Qualitative Analysis Workflows B.08.00 (Agilent, Palo Alto, CA, USA), and chromatographic peaks were integrated and corrected with MassHunter quantitative.

### Statistical analysis

After the metabolite data was transformed with Hellinger transformation, principal component analysis (PCA) was performed using the function rda in the R package vegan v 2.6-2 [41]. In addition, the data set that was log_10_ transform and mean centering were imported into the R package MetaboAnalystR v5.0 [42] to conduct orthogonal partial least squares-discriminant analysis (OPLS-DA) and extract the variable important in projection (VIP) value from the analysis results to evaluate the variance importance of compounds. The values of R2X, R2Y, and Q2 for OPLS-DA models were showed in Figure S2. In order to avoid overfitting, a permutation test (100 permutations) was performed. Based on Bray–Curtis’s dissimilarity distances of the composition and abundance of all volatile metabolites after ln transform, which were calculated using the function vegdist built in vegan, hierarchical clustering was visualized with the R package factoextra v.1.0.7 [43].

The chloroplast sequence alignments of *A. sinensis*, *A. dahurica*, *A. biserrata*, and *A. keiskei* were generated using MAFFT v7.475 [44]. Phylogenetic trees were constructed by maximum likelihood using IQ-TREE v 2.1.2 [45] with *Hydrocotyle sibthorpioides* Lam. as an outgroup.

All identified metabolites were annotated with KEGG database (http://www.kegg.jp/kegg/compound/) and further subjected to KEGG enrichment analyses with the R package clusterProfiler v. 4.4.4 [46].

## Supplementary Information

Supplementary Material 1. Figure S1: TIC chromatogram of quality control samples; Figure S2: Permutation test of OPLS-DA model; Figure S3: The violin plot of relative abundance of 15 classes in the four *Angelica* species.

Supplementary Material 2. Table S1: The volatile metabolites detected; Table S2: The *p*-values of the t-test for 15 classes in four species.

## Data Availability

The data presented in this study are available on request from the corresponding author.
