# Supplementary material for: Widely targeted metabolomics analysis reveals differences in volatile metabolites among four Angelica species
Source: Nat Prod Bioprospect. 2025 Jan 2;15(1):2. doi: 10.1007/s13659-024-00485-5 (PMC11693638; doi:10.1007/s13659-024-00485-5)
Supplement: Supplementary file 1 — Supplementary Material 1. Figure S1: TIC chromatogram of quality control samples; Figure S2: Permutation test of OPLS-DA model; Figure S3: The violin plot of relative abundance of 15 classes in the four Angelica species. [file 13659_2024_485_MOESM1_ESM.docx]

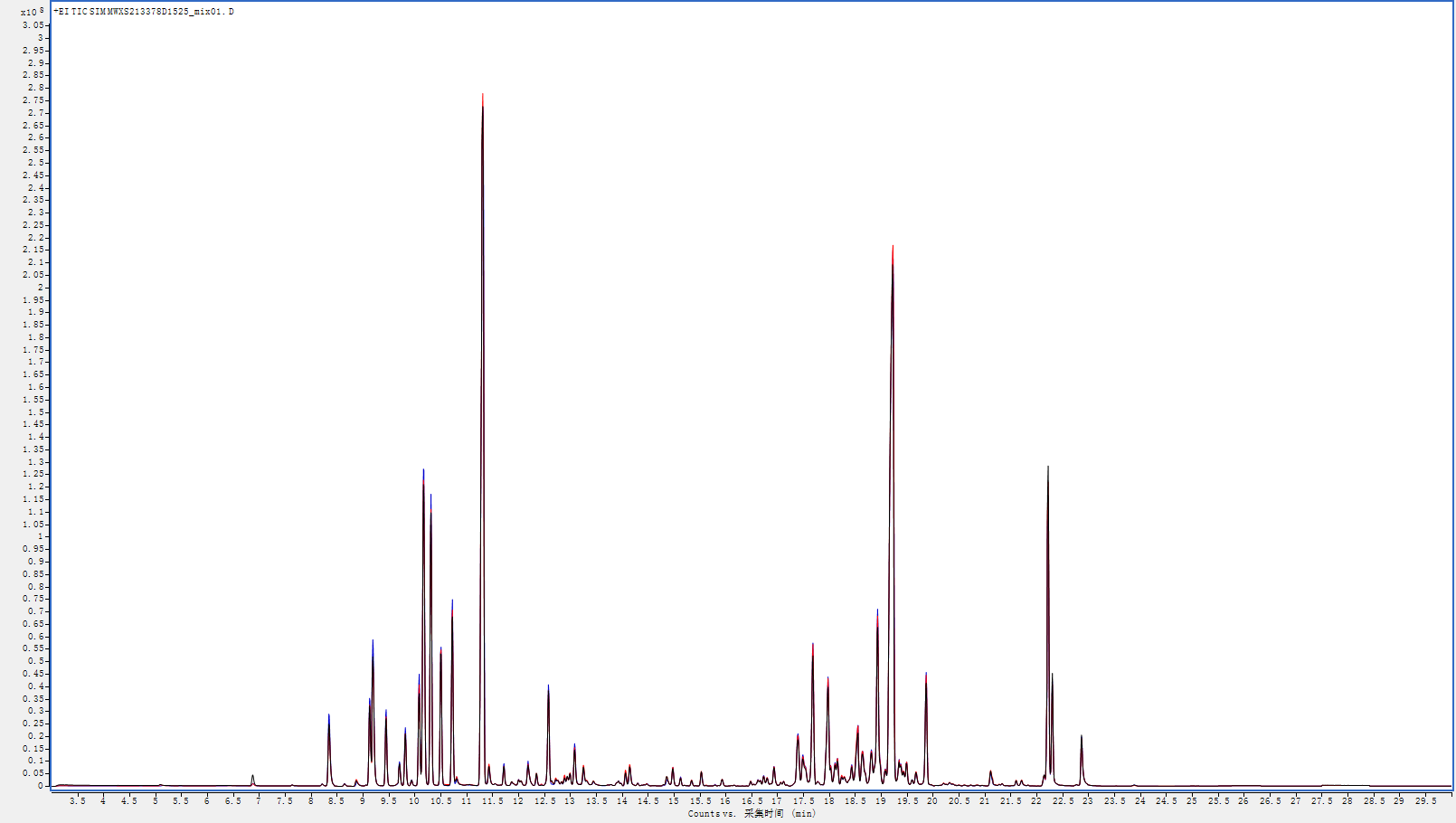


Figure S1. TIC chromatogram of quality control samples. The color indicates the number of quality control repetitions.


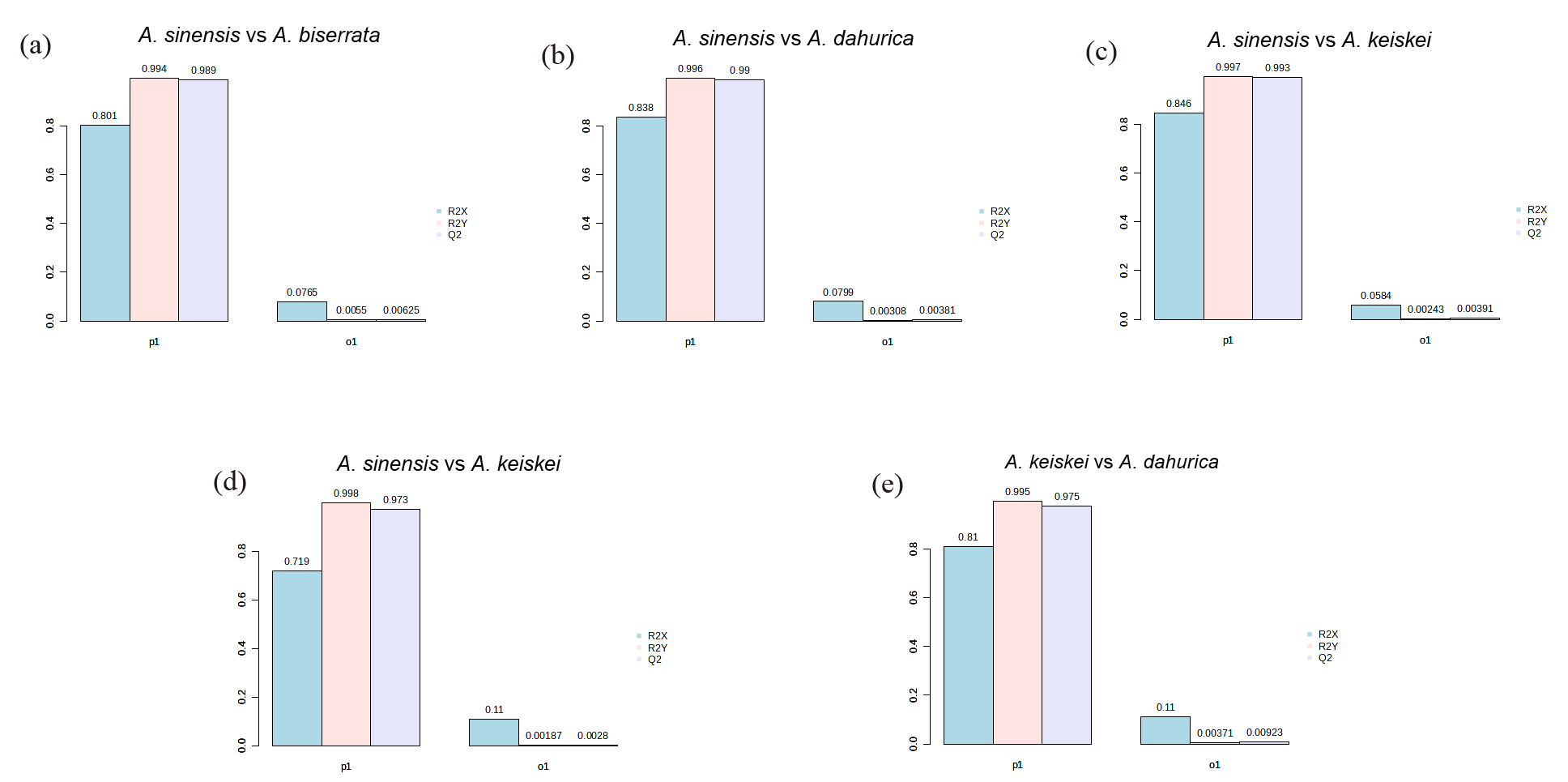


Figure S2. The values of R2X, R2Y, and Q2 for OPLS-DA models.


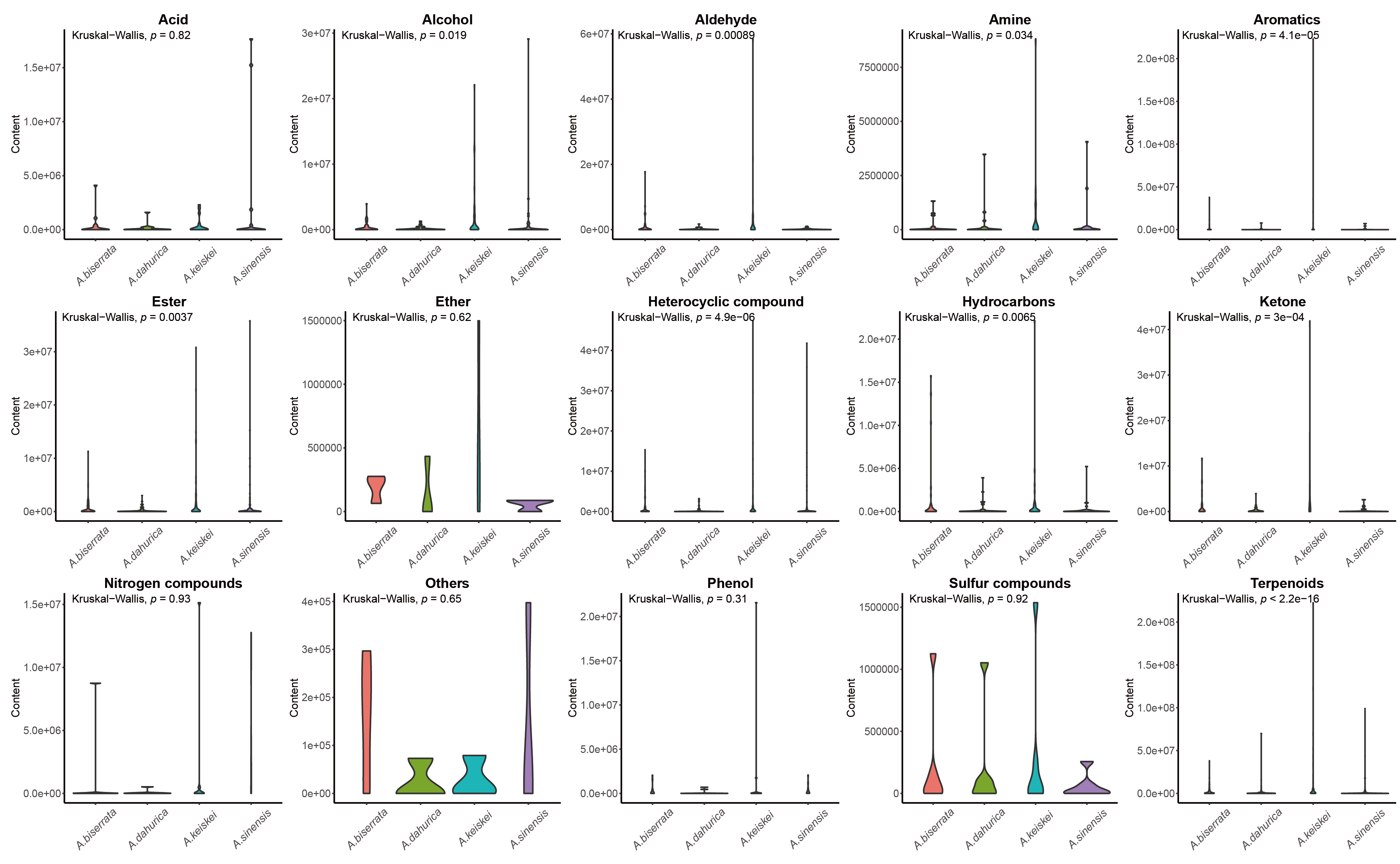


Figure S3. The violin plot of relative abundance of 15 classes in the four *Angelica* species.
